# 613. The Clinical Spectrum of *Curvularia* Species: A 20-year Experience at 2 Tertiary Care Hospitals

**DOI:** 10.1093/ofid/ofad500.679

**Published:** 2023-11-27

**Authors:** Jessica S Little, Eliezer Zachary Nussbaum, Joseph Braidt, Sanjat Kanjilal, Sarah P Hammond

**Affiliations:** Brigham and Women's Hospital, Boston, Massachusetts; Tufts Medical Center/Tufts University School of Medicine/Division of Geographic Medicine and Infectious Disease, Cambridge, Massachusetts; Massachusetts General Hospital, Boston, Massachusetts; Department of Population Medicine, Harvard Medical School and Harvard Pilgrim Healthcare Institute, Cambridge, MA; Massachusetts General Hospital, Boston, Massachusetts

## Abstract

**Background:**

*Curvularia* species are dematiaceous environmental molds that rarely cause disease in humans. Infections may be superficial or invasive and can present as disseminated infection or localized infection including keratitis, invasive fungal sinusitis (IFS), and skin and soft tissue infection (SSTI). Non-pathogenic colonization is also common. The frequency and spectrum of infection has not been well characterized.

**Methods:**

This was a retrospective study of all *Curvularia* infections at two institutions in Boston, MA between January 1, 2002 and January 1, 2023. Infections were identified using microbiologic culture databases and a research patient data registry. Clinical information was collected. This study was approved by the Massachusetts General Brigham IRB; research was conducted in accordance with the Declaration of Helsinki.

**Results:**

*Curvularia* species were identified from clinical specimens in 36 patients: 28 pathogenic infections and 8 cases of non-pathogenic colonization. Clinical characteristics of the pathogenic infections are described in Table 1. Of those with infection, 5 were invasive (2 disseminated; 3 localized) and occurred only in immunocompromised hosts (hematologic malignancy n=4, heart transplant n=1). Local invasive disease was characterized by SSTI (n=2) and IFS (n=1). There were 6 SSTI or wound infections without correlative pathology demonstrating tissue invasive disease (3 treated with triazoles; 3 not treated). All cases were identified by culture except one case identified by 28s ribosomal DNA PCR testing. Polymicrobial infection was common with > 50% of pathogenic infections exhibiting cultures with additional bacterial and/or fungal organisms. Antifungal susceptibility testing was performed in most invasive infections; results are shown in Table 2. Invasive infections were treated with both single and dual-agent antifungal regimens (posaconazole n=3; voriconazole and terbinafine n=1; voriconazole and liposomal amphotericin n=1).

**Figure:**
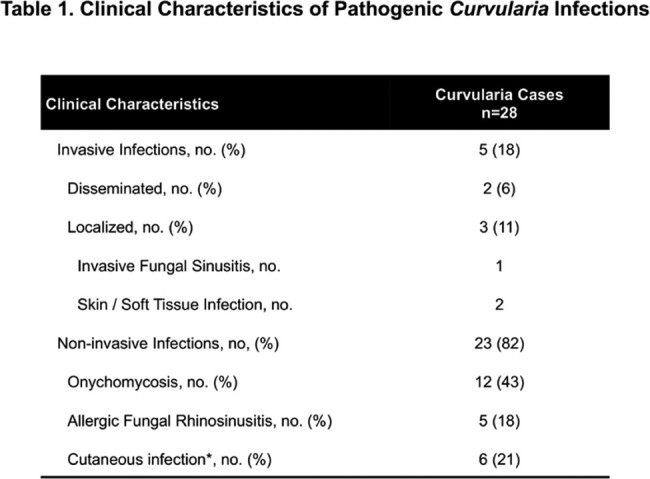

**Figure:**
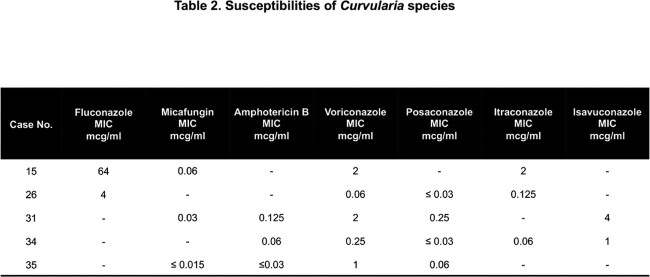

**Conclusion:**

Invasive *Curvularia* infections are rare but mainly occur the immunocompromised, and here manifested as disseminated disease, IFS, and SSTI. Coinfection is common. Non-invasive infections are more frequent (82%) and occur in normal hosts.

**Disclosures:**

**Sanjat Kanjilal, MD, MPH**, GSK: Advisor/Consultant|Pattern biosciences: Advisor/Consultant|Roche: Honoraria|Uptodate: Royalties **Sarah P. Hammond, MD**, F2G: Advisor/Consultant|F2G: Grant/Research Support|GSK: Grant/Research Support|Pfizer: Advisor/Consultant|Scynexis: Grant/Research Support|Seres therapeutics: Advisor/Consultant

